# Behavior data of battery and battery pack SOC estimation under different working conditions

**DOI:** 10.1016/j.dib.2016.10.012

**Published:** 2016-10-25

**Authors:** Xu Zhang, Yujie Wang, Duo Yang, Zonghai Chen

**Affiliations:** Department of Automation, University of Science and Technology of China, Hefei 230027, PR China

## Abstract

This article provides the dataset of operating conditions of battery behavior. The constant current condition and the dynamic stress test (DST) condition were carried out to analyze the battery discharging and charging features. The datasets were achieved at room temperature, in April, 2016. The shared data contributes to clarify the battery pack state-of-charge (SOC) and the battery inconsistency, which is also shown in the article of “An on-line estimation of battery pack parameters and state-of-charge using dual filters based on pack model” (X. Zhang, Y. Wang, D. Yang, et al., 2016) [1].

**Specifications Table**

**Table:** 

Subject area	*Energy*
More specific subject area	*Electric Vehicles*
Type of data	*Table, Matlab figures, Excel file, text file*
How data was acquired	*Battery performance test experiments (laboratory)*
Data format	*Raw, analyzed (processed).*
Experimental factors	*At room temperature*
Experimental features	*Batteries were charged by a programmable DC power supply, Chroma 62006P-30–80, and were discharged with an electrical load. The sample time was 1 s.*
Data source location	*University of Science and Technology of China, Hefei, China*
Data accessibility	*Data is within this article*

**Value of the data**

• We show the data of three batteries’ capacity, which can be used for lithium-ion battery SOC estimation.
• The data can be used to analyze the performance of lithium-ion battery and identify the battery parameters.
• The data under the constant current condition and DST condition can be used to analyze the dynamic behavior of battery pack.

## 1 Data

The shared data describes the behavior of single cell and battery pack under the constant current condition and the DST [2] condition at room temperature (25 °C) in April, 2016. Contents include every cell voltage, the total voltage of the battery pack, the load current (we defined that the load current was negative when the battery was discharged,or else the load current was positive in Appendix A), and the sampling time.

Those data can be used to analyze the dynamic behavior of battery and also can estimate the battery SOC.

## 2 Experimental design, materials and methods

In order to test the battery performance, a DC power supply is needed for charging and an electrical load is needed for discharging. The DC power supply we used is Chroma 62006P-30–80, which is programmable. A PC is used for data record and storage. All the tests were experimented in 25 °C.

### 2.1 The capacity of batteries

The type of the LiFePO_4_ battery we used is IFP1865140.The experiment was designed as follows: a CC–CV (constant current–constant voltage) charging experiment was carried out when the battery was completely empty to obtain the current maximum available capacity of a battery. This test was carried out three times in order to make the data reliable. In the experiments, there are three batteries and the capacities were shown in Table 1.

Table 1 shows the information extracted from the original data of 3 batteries. The state-of-charge (SOC) was calculated by Eq. (1).

(1) $$SOC(t)=SOC(t_{0})+\eta_{c}\int_{t_{0}}^{t}i(\tau )d\tau /C_{n}$$

### 2.2 The relationship between SOC and open circuit voltage (OCV)

**Fig. 1:**
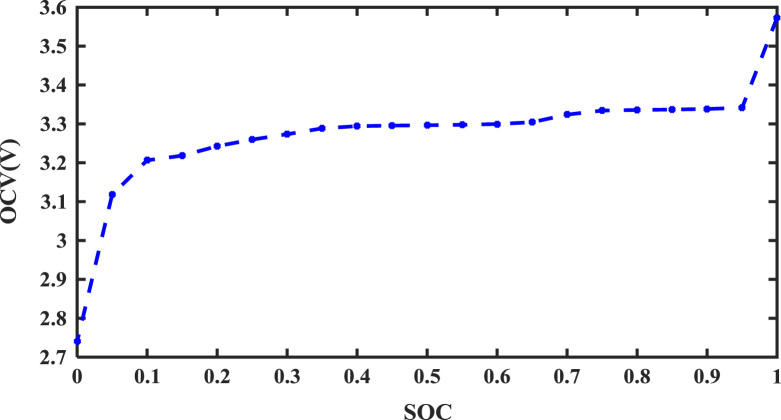
The relationship between SOC and OCV.

Before this experiment, the battery was firstly fully charged. At every working condition, the battery was discharged with 1/2 rate of current for 12 min and then the battery was rested for an hour until the battery was fully discharged. The relationship between the SOC and OCV was shown in Fig. 1.

### 2.3 The behavior of battery pack

In order to reflect the dynamic behavior of battery pack and estimate the battery pack SOC (the battery pack included three batteries connected by three series and one parallel), we did the experiments as follows:

1) The three batteries were fully charged, and then the battery pack was discharged under constant 1C current until the battery pack was fully discharged.
2) The three batteries were fully charged, and then the battery pack was discharged under DST condition until the battery pack was fully discharged.
3) The initial SOC of cell1 is 1, cell2 is 0.8, cell3 is 0.6, and then the battery pack was discharged under DST condition until one of the cells’ voltage reached the cut-off voltage.

## Figures and Tables

**Table 1:** The capacity of three batteries.

Cell number	First (AH)	Second (AH)	Third (AH)	Average (AH)
1	9.565	9.562	9.545	9.557
2	9.604	9.590	9.584	9.594
3	9.529	9.543	9.540	9.537

## Footnotes

Appendix A Supplementary data associated with this article can be found in the online version at doi:10.1016/j.dib.2016.10.012.
