# Supplementary material for: Behavior data of battery and battery pack SOC estimation under different working conditions
Source: Data Brief. 2016 Oct 25;9:737–40. doi: 10.1016/j.dib.2016.10.012 (PMC5094688; doi:10.1016/j.dib.2016.10.012)
Supplement: Supplementary file 2 — Supplementary material [file mmc2.zip › Appendix A. Supporting information/mmc4.docx]

Dear editor(s) of DIB,

Attached, please find our manuscript entitled “Behavior data of battery and battery pack SOC estimation under different working conditions”.

This article provides the dataset of operating conditions of battery behavior. The constant current condition and the dynamic stress test condition were carried out to analyze the battery discharging and charging features. The datasets were achieved at room temperature, in April, 2016. The shared data contributes to clarify the battery pack state-of-charge and the battery inconsistency.

The authors whose names are listed immediately below certify that they have NO affiliations with or involvement in any organization or entity with any financial interest (such as honoraria; educational grants; participation in speakers’ bureaus; membership, employment, consultancies, stock ownership, or other equity interest; and expert testimony or patent-licensing arrangements), or non-financial interest (such as personal or professional relationships, affiliations, knowledge or beliefs) in the subject matter or materials discussed in this manuscript.

We the undersigned declare that this manuscript is original, has not been published before and is not currently being considered for publication elsewhere.

We wish to confirm that there are no known conflicts of interest associated with this publication and there has been no significant financial support for this work that could have influenced its outcome.

We confirm that the manuscript has been read and approved by all named authors and that there are no other persons who satisfied the criteria for authorship but are not listed.

We further confirm that the order of authors listed in the manuscript has been approved by all of us.

We confirm that we have given due consideration to the protection of intellectual property associated with this work and that there are no impediments to publication, including the timing of publication, with respect to intellectual property. In so doing we confirm that we have followed the regulations of our institutions concerning intellectual property.

We understand that the Corresponding Author is the sole contact for the Editorial process (including Editorial Manager and direct communications with the office). He is responsible for communicating with the other authors about progress, submissions of revisions and final approval of proofs.

We confirm that we have provided a current, correct email address which is accessible by the Corresponding Author and which has been configured to accept email from Elsevier DIB department.

Xu Zhang, PhD candidate

Department of Automation, University of Science and Technology of China, Hefei, PR China.

**Signature:**

**Date:** April 19, 2016

Yujie Wang, PhD candidate

Department of Automation, University of Science and Technology of China, Hefei, PR China.

**Signature:**

**Date:** April 19, 2016

Duo Yang, M.A

Department of Automation, University of Science and Technology of China, Hefei, PR China.

**Signature:**

**Date:** April 19, 2016

Zonghai Chen, Professor

Department of Automation, University of Science and Technology of China, Hefei, PR China.

**Signature: *on behalf of Professor Chen***

**Date:** April 19, 2016

Corresponding Author: Professor Zonghai Chen, [chenzh@ustc.edu.cn](mailto:chenzh@ustc.edu.cn); Address: Department of Automation, University of Science and Technology of China, Hefei, 230027, PR China. Tel: +86 055163606104
